# Co-Delivery of D-LAK Antimicrobial Peptide and Capreomycin as Inhaled Powder Formulation to Combat Drug-Resistant Tuberculosis

**DOI:** 10.1007/s11095-023-03488-y

**Published:** 2023-03-03

**Authors:** Zitong Shao, Michael Y. T. Chow, Shing Fung Chow, Jenny K. W. Lam

**Affiliations:** 1 Department of Pharmacology and Pharmacy, Li Ka Shing Faculty of Medicine, The University of Hong Kong, Pokfulam, Hong Kong SAR; 2 Department of Pharmaceutics, UCL School of Pharmacy, University College London, London, UK; 3 Advanced Biomedical Instrumentation Centre, Hong Kong Science Park, Shatin, New Territories Hong Kong SAR

**Keywords:** antimicrobial peptide, capreomycin, drug-resistant tuberculosis, dry powder aerosol, spray drying

## Abstract

**Introduction:**

The emergence of multidrug-resistant (MDR) *Mycobacterium tuberculosis* (*Mtb*) posed a severe challenge to tuberculosis (TB) management. The treatment of MDR-TB involves second-line anti-TB agents, most of which are injectable and highly toxic. Previous metabolomics study of the *Mtb* membrane revealed that two antimicrobial peptides, D-LAK120-A and D-LAK120-HP13, can potentiate the efficacy of capreomycin against mycobacteria.

**Aims:**

As both capreomycin and peptides are not orally available, this study aimed to formulate combined formulations of capreomycin and D-LAK peptides as inhalable dry powder by spray drying.

**Methods and Results:**

A total of 16 formulations were prepared with different levels of drug content and capreomycin to peptide ratios. A good production yield of over 60% (w/w) was achieved in most formulations. The co-spray dried particles exhibited spherical shape with a smooth surface and contained low residual moisture of below 2%. Both capreomycin and D-LAK peptides were enriched at the surface of the particles. The aerosol performance of the formulations was evaluated with Next Generation Impactor (NGI) coupled with Breezhaler®. While no significant difference was observed in terms of emitted fraction (EF) and fine particle fraction (FPF) among the different formulations, lowering the flow rate from 90 L/min to 60 L/min could reduce the impaction at the throat and improve the FPF to over 50%.

**Conclusions:**

Overall, this study showed the feasibility of producing co-spray dried formulation of capreomycin and antimicrobial peptides for pulmonary delivery. Future study on their antibacterial effect is warranted.

**Supplementary Information:**

The online version contains supplementary material available at 10.1007/s11095-023-03488-y.

## Introduction

Tuberculosis (TB) is an airborne communicable disease caused by the infection of bacillus *Mycobacterium tuberculosis* (*Mtb*), affecting primarily the lung but may spread to the bone and central nervous system [1]. According to the latest estimates of global mortality by cause, TB is ranked as the top leading cause of death from a single infectious agent in 2019 [2]. The pandemic of coronavirus disease 2019 (COVID-19) threatens global TB control further through restricting the access to TB diagnosis and treatment, specifically in the treatment of drug-resistant TB [3]. TB that is resistant to the most effective first-line drugs, namely isoniazid and rifampicin, remains an alarming concern. The treatment of drug-resistant TB often requires a course of at least 9 months and up to 20 months of second-line drugs [3] which are usually more toxic and expensive, and parenteral injections are often required. The frequent injection of the second-line agents (for instance, capreomycin is injected five times per week), their long treatment course and severe systemic adverse effects have led to poor patient compliance.

The treatment success rate for multi-drug resistant (MDR) TB (resistance to at least both isoniazid and rifampicin [4]) and rifampicin-resistant TB was only around 59% according to the latest available data in 2018 [3], despite the rate being steadily increased from 50% in 2012. The development of a shorter, safer, and more effective TB therapy, particularly against MDR-TB, remains in urgent demand. In the search for novel anti-TB therapeutics in combating MDR-TB, antimicrobial peptides (AMPs) are regarded as auspicious candidates due to their ability to inhibit the growth of *Mtb* with multiple mechanisms and immunomodulation activity [5, 6]. AMPs are typically small, cationic, amphipathic peptides consisted of 12–50 amino acid residues with a broad spectrum activity against pathogens [7]. The mechanism of AMPs against *Mtb* was multifaceted. Active AMPs can interact with the mycobacterial cell envelope selectively, destabilize or penetrate the membrane, leading to the leakage of cellular fluid and finally killing the mycobacteria [8]. AMPs with intracellular nucleic acid or protein inhibition activity can also act as an immunostimulatory agent to exert antimicrobial function [5, 7, 8]. Because of the diverse yet direct and rapid bactericidal activities, it is more difficult for *Mtb* to develop resistance to AMPs [9].

The two D-LAK peptides used in this study, D-LAK120-A and D-LAK120-HP13, are synthetic analogues consisting of 25 _D_-enantiomer amino acid residues with a nominal charge of + 9 at neutral pH [7]. Unlike the endogenous AMPs, D-LAK peptides are in D-conformation that reduce their vulnerability to protease activity *in vivo* [5, 7]. D-LAK peptides with 120° charge angle demonstrated the highest potency against *Mtb* [7]. D-LAK120-HP13 also contains a kink-inducing proline, the incorporation of which in specific site of D-LAK peptide not only improves the antibacterial activity, but also mitigates the hemolytic effect [7, 10]. According to our previous findings, D-LAK120-A and D-LAK120-HP13 can act synergistically with capreomycin against *Mycobacterium smegmatis* [11]. Capreomycin, a semisynthetic antibiotic composed of four molecular analogs, is a second-line drug used in the treatment of isoniazid- and rifampicin-resistant TB [12]. It was found that capreomycin altered the metabolism and remodeled the cell envelope of *Mycobacterium smegmatis*, rendering the cell wall more rigid and resistant to antibiotics [11]. The presence of D-LAK120-A or D-LAK120-HP13 modified the surface properties of mycobacteria and mitigated the remodeling induced by capreomycin, therefore potentiating the efficacy of capreomycin *in vitro*.

To take advantage of this synergistic effect between capreomycin and D-LAK peptides, co-delivery of the two as a combined inhaled dosage form is proposed as a new strategy for treating MDR-TB. Neither capreomycin nor D-LAK peptides are orally available. Parenteral administration is not desirable due to its invasiveness and overall poor patient acceptance, especially MDR-TB treatment usually lasts for many months. Moreover, systemic administration may result in poor lung distribution [6], hence a high dose is required to achieve effective concentration at the site of infection, increasing the risk of toxicity. Pulmonary delivery is a promising approach for the treatment of TB owing to the advantages of ensuring high drug concentration at the infection site with reduced risk of systemic adverse effects. Moreover, inhaled dry powder formulation offers better stability, avoiding cold-chain logistics or reconstitution, which is crucial for developing countries. Our group has previously prepared inhalable dry powder of capreomycin and D-LAK peptide separately by spray drying [9, 13]. It is anticipated that the anti-TB activity of the D-LAK peptides can be maintained after spray drying, and their synergistic effect with capreomycin will be retained. This study aimed to take a step further with the focus on formulating capreomycin in combination with D-LAK peptide as inhalable dry powders by spray drying. The physicochemical characteristics and aerosol performance of the powders were thoroughly evaluated.

## Materials and Methods

### Materials

D-LAK120-A peptide (KKLALALAKKWLALAKKLALALAKK-NH_2_) and D-LAK120-HP13 peptide (KKALAHALKKWLPALKKLAHALAKK-NH_2_) with a purity over 80% were purchased from EZBiolab (Parsippany, USA). Mannitol (Pearlitol® 160) was obtained from Roquette (Lestrem, France). Capreomycin sulfate (Capastat sulfate) was purchased from Yick-Vic (Hong Kong). Analytical standard of capreomycin sulfate and trifluoroacetic acid (TFA) were purchased from Sigma-Aldrich (Poole, UK). All solvents and reagents were of analytical grade or better.

### Spray Drying of Capreomycin/D-LAK Peptide Formulations

Capreomycin, D-LAK peptides (D-LAK120-A or D-LAK120-HP13) and mannitol were dissolved in ultra-pure water at different mass ratios (Table I) to make a final solute concentration of 1% (w/v). The solutions were spray dried using a laboratory spray dryer (Mini Spray Dryer B-290, Büchi Labortechnik, Flawil, Switzerland) coupled with a high-performance cyclone under the following parameters: inlet temperature 80°C; nitrogen atomization flow rate 742 L/h; air aspiration 38 m^3^/min; liquid feed rate 2.1 mL/min. A two-fluid spray nozzle with an internal diameter of 0.7 mm (Büchi Labortechnik, Flawil, Switzerland) was used and the spray dryer was operated at the open-loop configuration. D-LAK120-A and D-LAK120-HP13 correspond to A series and B series, respectively. A total of 16 formulations were produced, with eight formulations for each peptide (Table I). The mass ratios of peptide to capreomycin (4:1 or 8:1, w/w) used in the formulations were determined according to their minimum inhibitory concentrations (MICs) that produced synergistic effect against *M. smegmatis mc*^*2*^ and *Mtb* Bleupan strains [4, 6]. The total drug content ranged from 2 to 20%.

**Table I:** The Composition of Co-Spray Dried Formulations Containing Capreomycin Sulfate (Cap), D-LAK Peptide and Mannitol

Sample	Peptide	Cap (%, w/w)	Peptide (%, w/w)	Mannitol (%, w/w)
A0	D-LAK120-A	Nil	2.0	98.0
A1		0.5	2.0	97.5
A2		0.5	4.0	95.5
A3		1.0	4.0	95.0
A4		1.0	8.0	91.0
A5		2.0	8.0	90.0
A6		2.0	16.0	82.0
A7		4.0	16.0	80.0
B0	D-LAK120-HP13	Nil	2.0	98.0
B1		0.5	2.0	97.5
B2		0.5	4.0	95.5
B3		1.0	4.0	95.0
B4		1.0	8.0	91.0
B5		2.0	8.0	90.0
B6		2.0	16.0	82.0
B7		4.0	16.0	80.0

### Production Yield and Drug Content Measurement

The production yield was calculated as the mass of powder collected after spray drying divided by the total mass of solid input. To measure the drug content in the co-spray dried powder, weighed powder was dissolved in ultra-pure water to prepare a 5 mL solution. The dissolved sample was filtered through a 0.45-µm nylon membrane filter and quantified with high-performance liquid chromatography (HPLC, Agilent 1260 Infinity, Agilent Technologies, Santa Clara, USA). The contents of capreomycin, peptide and mannitol were measured separately and were calculated as the measured amount with respect to the powder mass. The HPLC method for quantification of capreomycin was adopted and modified according to a previous study [14]. The mobile phase consisted of 0.1% TFA aqueous solution (pH = 2) and acetonitrile (95:5, v/v) was run at a rate of 1 mL/min for 10 min. Each sample containing 50 μL solution was injected and passed through the C18 column (5 μm, 250 mm, Agilent, USA) at room temperature, and the capreomycin was detected by UV at wavelength 268 nm. The capreomycin IIA/IIB eluted at approximately 4.6 min and capreomycin IA/IB at around 5.4 min. Linearity for total capreomycin was demonstrated between 5.0 μg/mL and 200 μg/mL (R^2^ = 0.9999). D-LAK peptides were quantified using an established HPLC method [9]. The mobile phase consisted of acetonitrile and ultra-pure water with 0.1% TFA. A linear gradient from 20 to 80% acetonitrile was applied over 20 min at 1 mL/min. Each sample containing 100 μL of solution was injected by an auto-sampler and passed through a C18 column (250 mm × 4.6 mm, 5 μm, VydacTM GraceTM, IL, USA) at ambient temperature. The peptide was detected by UV at wavelength 220 nm. The retention time of D-LAK peptides was around 9.85 min. Linearity for D-LAK peptides was demonstrated between 8.0 μg/mL and 200 μg/mL (R^2^ = 0.9998). The HPLC method for mannitol measurement was detailed in Sect. “Aerosol performance and quantification of mannitol”.

### Morphology Study

The morphology of the co-spray dried powders was visualized by scanning electron microscopy (SEM, Hitachi S-4800, Hitachi High-technologies Crop., Tokyo, Japan) at 5.0 kV. A small amount of powders was sprinkled on the adhesive carbon discs mounted on metal stubs. Excess powders were removed by blowing with clean compressed air. Prior to imaging, samples were sputter-coated with approximately 13 nm gold–palladium alloy in two cycles (60 s each) using a sputter coater (Q150T Plus Turbomolecular pumped coater, Quorum, UK) to aid charge dissipation during imaging.

### Particle Size Distribution

The volumetric size distribution of co-spray dried powders was measured by laser diffraction (HELOS/KR, Sympatec, Clausthal-Zellerfeld, Germany, incorporate with an inhaler module). Approximately 4 mg of powder was loaded in a hydroxypropyl methylcellulose (HPMC) capsule and dispersed by Breezhaler® at a flow rate of 60 L/min which corresponded to a pressure drop of 1.5 kPa. The 100 mm (R3) lens (measuring range 0.45–175 μm) was utilized in the measurement. Each powder formulation was analyzed in triplicate (n = 3). The tenth (D10), the median (D50), and the ninetieth (D90) percentile of the volumetric diameter were recorded. The span was calculated using the formula (D90–D10)/D50.

### Aerosol Performance and Quantification of Mannitol

The aerosol performance of the co-spray dried powder was evaluated with Next Generations Impactor (NGI) (Copley Scientific Limited. Nottingham, UK) without a pre-separator according to the British Pharmacopeia method [15]. Approximately 5 mg of powder was loaded in an HPMC capsule and aerosolized using Breezhaler® (Novartis Pharmaceuticals, Hong Kong). The dispersion was operated at an airflow rate of 90 L/min for 2.7 s, which corresponded to a pressure drop of 3.5 kPa [16]. For formulations A1 and B1, their performance was also evaluated at an additional airflow rate of 60 L/min for 4 s with a pressure drop of 1.5 kPa. Powder that remained in the capsule, inhaler, adaptor and deposited on each part of NGI was collected separately by dissolving the powder in 4 mL of ultra-pure water. Mannitol, the bulking excipient which has the highest composition in all formulations was quantified by HPLC (Agilent 1260 Infinity; Agilent Technologies, Santa Clara, USA) with a refractive index detector (G1362A, Agilent Technologies, Santa Clara, USA). For each sample, 50 μL of filtered solution was injected and eluted through two ion-exchange ligand-change columns (Agilent Hi-Plex Ca, 7.7 × 50 mm, 8 μm; Agilent Technologies), which was maintained at 75 ℃. The mobile phase was 100% ultra-pure water eluting at a flow rate of 0.6 mL/min. The total amount of powder recovered and assayed from the dispersion was referred as the recovered dose. Emitted fraction (EF) was defined as the amount of powder that exited the inhaler with respect to the recovered dose. Fine particle fraction (FPF) referred to the amount of powder with an aerodynamic diameter less than 5.0 μm (calculated by interpolation) with respect to the recovered dose. The mass median aerodynamic diameter (MMAD) was defined as the aerodynamic diameter at which 50% of the particles by mass are larger and 50% are smaller. The MMAD and geometric standard deviation (GSD) were obtained by a linear plot of cumulative mass percentage against the logarithm of the cut-off diameters.

### Thermogravimetric Analysis (TGA)

The residual moisture of co-spray dried powders was measured by thermogravimetric analysis (TGA) (TGA550, TA Instruments, New Castle, DE, USA). The loss in mass was monitored as the samples were heated from 25℃ to 160℃ at 10 ℃/min with 60 mL/min nitrogen purge. The loss in mass indicated the residual moisture that evaporated from the samples.

### Differential Scanning Calorimetry (DSC)

The thermal response profiles of co-spray dried powders and raw materials were assessed by differential scanning calorimetry (DSC) (Q1000, TA Instruments, New Castle, DE, USA). Approximately 3 mg of powder was loaded into an aluminum crucible and heated from 25℃ to 250℃ at a constant rate of 10 ℃/min under 50 mL/min nitrogen purge. The thermogram of each sample was obtained.

### Powder X-ray Diffraction (PXRD)

X-ray diffractogram was obtained using an X-ray diffractometer (Philips X’Pert PRO, PANalytical, Almelo, The Netherlands) to study the crystallinity of the co-spray dried powders. Sample was packed in custom-made aluminum holder with a 2 mm depth. The powder compact was measured with Cu-Kα radiation (λ = 1.5406 Å) at 40 mA and 40 kV, with an angular increment of 0.04° at 2 s per step, covering a 2 θ range from 5° to 50°.

### X-ray Photoelectron Spectroscopy (XPS)

The surface composition of the co-spray dried powders was investigated using an AXIS Ultra DLD Spectrometer (Kratos Analytical Ltd. Manchester UK). The monochromatic AI Kα X-ray at 150 W was employed. The pass energy and step sizes of survey scans were 160 eV and 1 eV. The pass energy and step sizes of snapshot scans were 40 eV and 0.1 eV, respectively. The pressure in analysis chamber during measurement was less than 5 × 10^−8^ Pa. One spot on each sample was randomly selected for measurement with a spot size of 700 × 300 µm (hybrid mode). The surface composition of each component in the powder sample was calculated (Supplementary Information 1.1).

### Statistical Analysis

The difference in aerosol performance (including EF, FPF and MMAD) of all formulations evaluated under the same condition (Breezhaler®, 90 L/min) was analyzed by one-way ANOVA followed by Tukey post hoc test by GraphPad Prism (version 8.0.1, GraphPad Software, San Diego, CA, USA). For the aerosol performance evaluated using Breezhaler® at two different flow rates (formulation A1 and B1), Student’s t-test was applied for comparison.

## Results

### Production Yield, Drug Content and Residual Moisture

At the current spray drying condition, the resultant outlet temperature ranged from 43 to 49 ℃. The production yield decreased with increasing drug content (Table II). For formulations containing D-LAK120-A, all except A6 and A7 had a good production yield of over 70%. A6 and A7 with high total drug content of 18% and 20%, respectively, had a low yield of below 50%. For formulations containing D-LAK120-HP13, a similar trend was observed. The production yield of B0 to B5 was above 60%, whereas the yield of B6 and B7 dropped to below 30%. The measured percentage of capreomycin and D-LAK peptides approximated the feed quantity for all formulations (Table II). Low residual water content of below 2% (w/w) was observed for all formulations, indicating that the spray drying condition employed in this study was effective for preparing dry powder of capreomycin and D-LAK peptide combination.

**Table II:** The Production Yield, Drug Content and Residual Moisture of Co-Spray Dried Powder Formulations. Data for Measured Percentage Presented as Mean ± Standard Deviation (n = 3). Cap—Capreomycin (Sulfate). *N.A. (Not Applicable)—the Weight Loss was Lower than the Detection Limit

Sample	Peptide	Production yield (%, w/w)	Measured percentage by mass			Residual moisture (%, w/w)
			Cap	Peptide	Mannitol	
A0	D-LAK120-A	79.00	Nil	2.04 ± 0.04	97.96 ± 0.04	N.A.*
A1		75.76	0.53 ± 0.01	1.92 ± 0.04	97.55 ± 0.04	0.21
A2		72.65	0.53 ± 0.01	3.72 ± 0.05	95.75 ± 0.05	0.08
A3		72.69	0.90 ± 0.06	3.90 ± 0.18	95.20 ± 0.22	0.55
A4		70.73	0.94 ± 0.02	7.98 ± 0.08	91.08 ± 0.10	0.63
A5		70.93	2.03 ± 0.04	7.31 ± 0.22	90.66 ± 0.21	0.90
A6		33.41	1.86 ± 0.19	16.54 ± 0.69	81.60 ± 0.82	1.86
A7		45.13	4.61 ± 0.11	16.41 ± 0.39	78.99 ± 0.40	1.27
B0	D-LAK120-HP13	76.81	Nil	1.76 ± 0.54	98.24 ± 0.54	0.34
B1		76.23	0.50 ± 0.02	1.57 ± 0.13	97.93 ± 0.15	0.09
B2		76.33	0.53 ± 0.02	3.50 ± 0.25	95.97 ± 0.27	0.38
B3		76.78	1.05 ± 0.02	4.04 ± 0.32	94.91 ± 0.31	0.47
B4		63.74	0.93 ± 0.01	7.50 ± 0.12	91.57 ± 0.13	0.51
B5		61.51	2.07 ± 0.02	7.19 ± 0.04	90.74 ± 0.05	1.04
B6		26.13	1.81 ± 0.16	16.92 ± 0.53	81.27 ± 0.67	N.A.*
B7		22.05	4.57 ± 0.28	15.23 ± 0.90	80.19 ± 0.95	0.78

### Particle Morphology

**Fig. 1:**
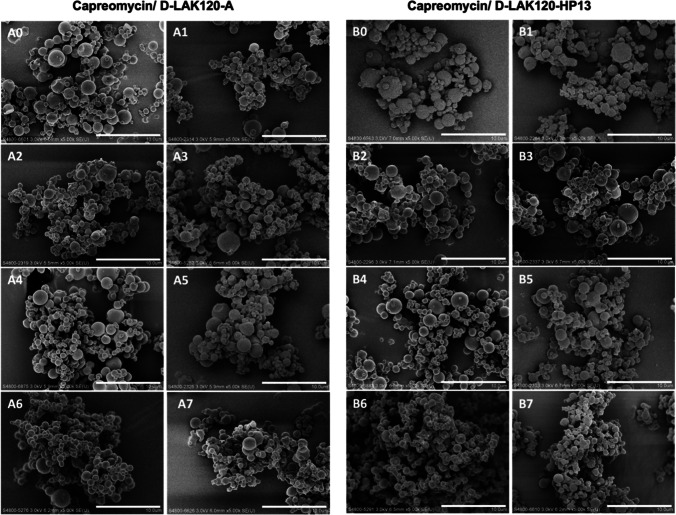
The scanning electron microscopy (SEM) images of co-spray-dried powder formulations at 5,000 magnification (scale bar = 10 μm).

As observed in the SEM images (Fig. 1), most of the co-spray dried particles exhibited spherical shape with a smooth surface. Partial aggregation was observed. Most of the particles were below 5 μm with the majority ranged between 2 to 3 μm. There was no obvious notable difference in the overall morphology of particles among different formulations.

### Particle Size Distribution and Aerosol Performance

**Fig. 2:**
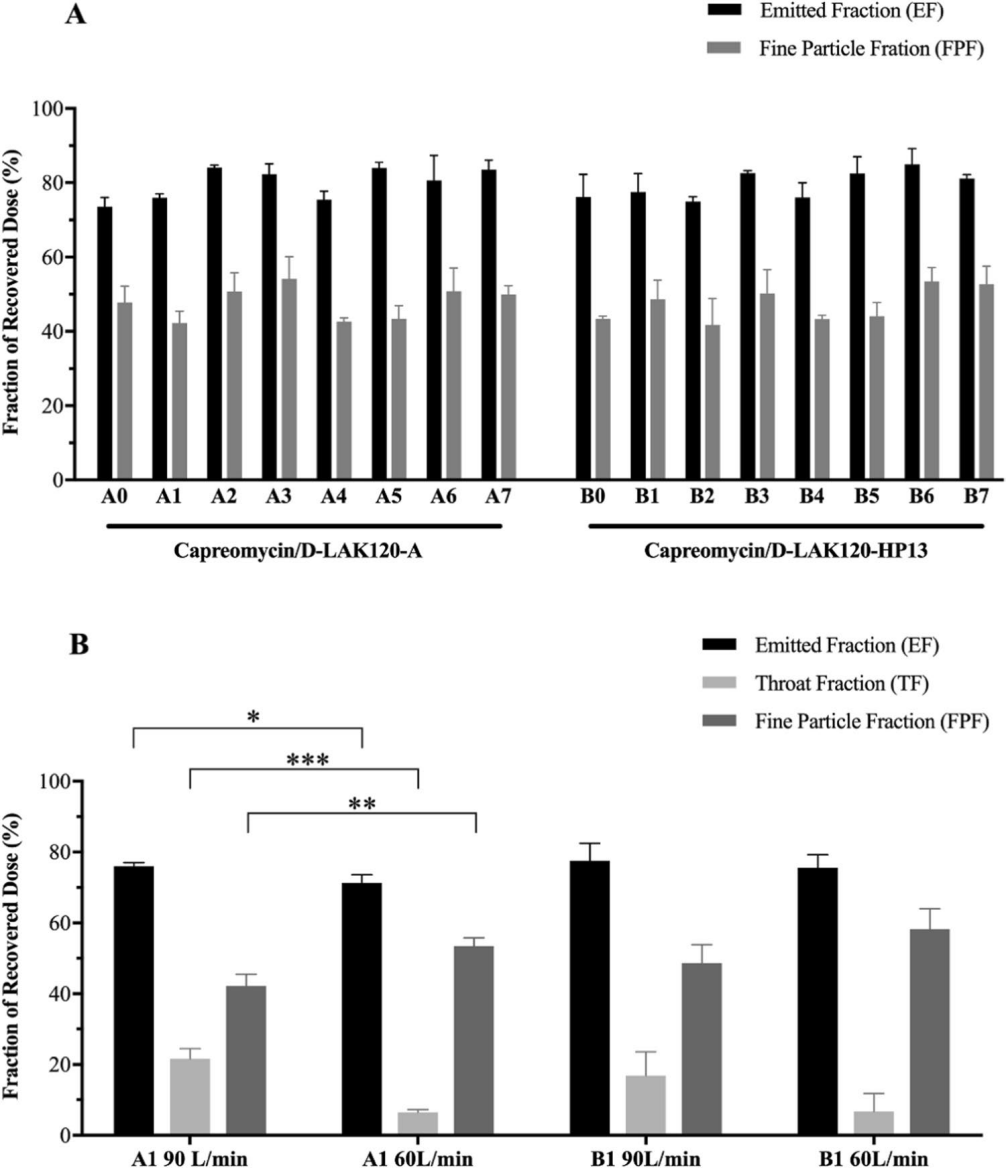
Aerosol performance of co-spray dried powder formulations evaluated by Next Generation Impactor (NGI) and the powder were dispersed with Breezhaler®. (**A**) The formulations were evaluated at a flow rate of 90 L/min. One-way ANOVA followed by Tukey post hoc test was applied; (**B**) A1 and B1 formulations were evaluated and compared at flow rates of 90 L/min and 60 L/min. Student’s t-test was applied for comparison. All data were presented as mean ± standard deviation (n = 3). *p*-value indicated comparison of EF, TF and FPF between the same formulation with different flow rate. *, **, *** represents *p* < 0.05, 0.01 and 0.001, respectively.

The volumetric diameter was measured for all formulations except A6, A7, B6 and B7 (there was insufficient material due to low production yield) (Table III). All measured formulations had a small particle size with D_50_ in the range of 2.4 to 3.3 μm, which was consistent with the SEM images. The aerosol performance of the powders was evaluated with NGI operated at a flow rate of 90 L/min (Fig. 2A). All formulations exhibited similar aerosol characteristics with EF ranged between 70 and 85% and FPF ranged between 40 and 55%. There was no significant difference in terms of EF and FPF among the formulations (p > 0.05, one-way AVONA). The MMAD of all formulations ranged from 4.1 to 6.2 μm (Table III). Formulation A2, A3, A6, A7, B1, B3, B6 and B7 exhibited MMAD below 5 μm. There was no noticeable correlation between the aerosol performance and the total drug content, nor the type of peptide involved. It was notable that more than 15% (with respect to the recovered dose by mass) of powder undesirably deposited at the throat of NGI in most formulations when the powder was dispersed at a high flow rate of 90 L/min (Supplementary Fig. S1). To further study the aerosol performance, NGI operated at a lower flow rate of 60 L/min was used to evaluate formulations A1 and B1. The EF, throat fraction (TF, defined as the amount of powder that deposited at the throat/ induction port of NGI with respect to the recovered dose), and FPF of these two formulations at different flow rates were presented (Fig. 2B). For formulation A1, when the flow rate was reduced from 90 L/min to 60 L/min, the EF decreased from 76 to 71% (p < 0.05) while the FPF increased from 42 to 53% (p < 0.01). There was a prominent change in TF which decreased from 22 to 7% (p < 0.001). While formulation B1 followed a similar trend, there was no significant difference in EF, TF and FPF when the powders were dispersed at 60 L/min and 90 L/min. Overall, dispersion of powder at a lower flow rate appeared to improve the aerosol performance of the co-spray dried powder formulations.

**Table III:** Particle Size Distribution of Co-Spray Dried Powder Formulations. The Volumetric Diameter was Obtained from Laser Diffraction Measurement (Breezhaler®, 60 L/min). The Median Aerodynamic Diameter (MMAD) and Geometric Standard Deviation (GSD) was Obtained from Next Generation Impactor Experiment (Breezhaler®, 90 L/min). Data for Volumetric Diameter and Aerodynamic Size were Presented as Mean ± Standard Deviation (n = 3). *Due to the Low Production Yield_,_ the Volumetric Diameter of A6, A7, B6 and B7 was not Measured

Sample	Volumetric diameter				Aerodynamic diameter	
	D_10_(μm)	D_50_(μm)	D_90_(μm)	Span value	MMAD(μm)	GSD
A0	1.26 ± 0.06	2.79 ± 0.03	5.51 ± 0.05	1.53 ± 0.06	5.12 ± 0.58	3.23 ± 0.20
A1	1.53 ± 0.04	3.00 ± 0.05	5.64 ± 0.06	1.37 ± 0.03	5.85 ± 0.25	2.70 ± 0.15
A2	1.36 ± 0.01	3.19 ± 0.03	6.77 ± 0.10	1.70 ± 0.02	4.79 ± 0.52	2.99 ± 0.26
A3	1.52 ± 0.02	2.87 ± 0.02	4.67 ± 0.05	1.10 ± 0.01	4.34 ± 0.46	2.63 ± 0.23
A4	1.11 ± 0.09	2.51 ± 0.01	4.95 ± 0.18	1.53 ± 0.01	6.09 ± 0.28	3.98 ± 0.43
A5	1.42 ± 0.08	3.14 ± 0.04	6.53 ± 0.06	1.62 ± 0.04	5.96 ± 0.62	3.26 ± 0.55
A6*	N.A	N.A	N.A	N.A	4.29 ± 0.34	2.15 ± 0.31
A7*	N.A	N.A	N.A	N.A	4.95 ± 0.24	3.98 ± 0.43
B0	1.45 ± 0.01	2.89 ± 0.02	5.44 ± 0.07	1.38 ± 0.03	6.04 ± 0.19	3.93 ± 0.22
B1	1.37 ± 0.05	2.91 ± 0.05	5.73 ± 0.09	1.50 ± 0.02	4.99 ± 0.65	2.86 ± 0.54
B2	1.23 ± 0.04	2.69 ± 0.04	5.30 ± 0.13	1.51 ± 0.05	5.99 ± 0.80	3.06 ± 0.52
B3	1.16 ± 0.06	2.69 ± 0.02	5.54 ± 0.14	1.63 ± 0.08	4.96 ± 0.80	3.20 ± 0.33
B4	1.05 ± 0.01	2.40 ± 0.12	4.73 ± 0.31	1.53 ± 0.03	5.73 ± 0.23	3.66 ± 0.09
B5	1.24 ± 0.04	3.22 ± 0.05	7.18 ± 0.16	1.85 ± 0.06	5.65 ± 0.52	2.66 ± 0.24
B6*	N.A	N.A	N.A	N.A	4.27 ± 0.33	2.31 ± 0.23
B7*	N.A	N.A	N.A	N.A	4.36 ± 0.47	3.16 ± 0.27

### Thermoanalysis and Powder Crystallinity

**Fig. 3:**
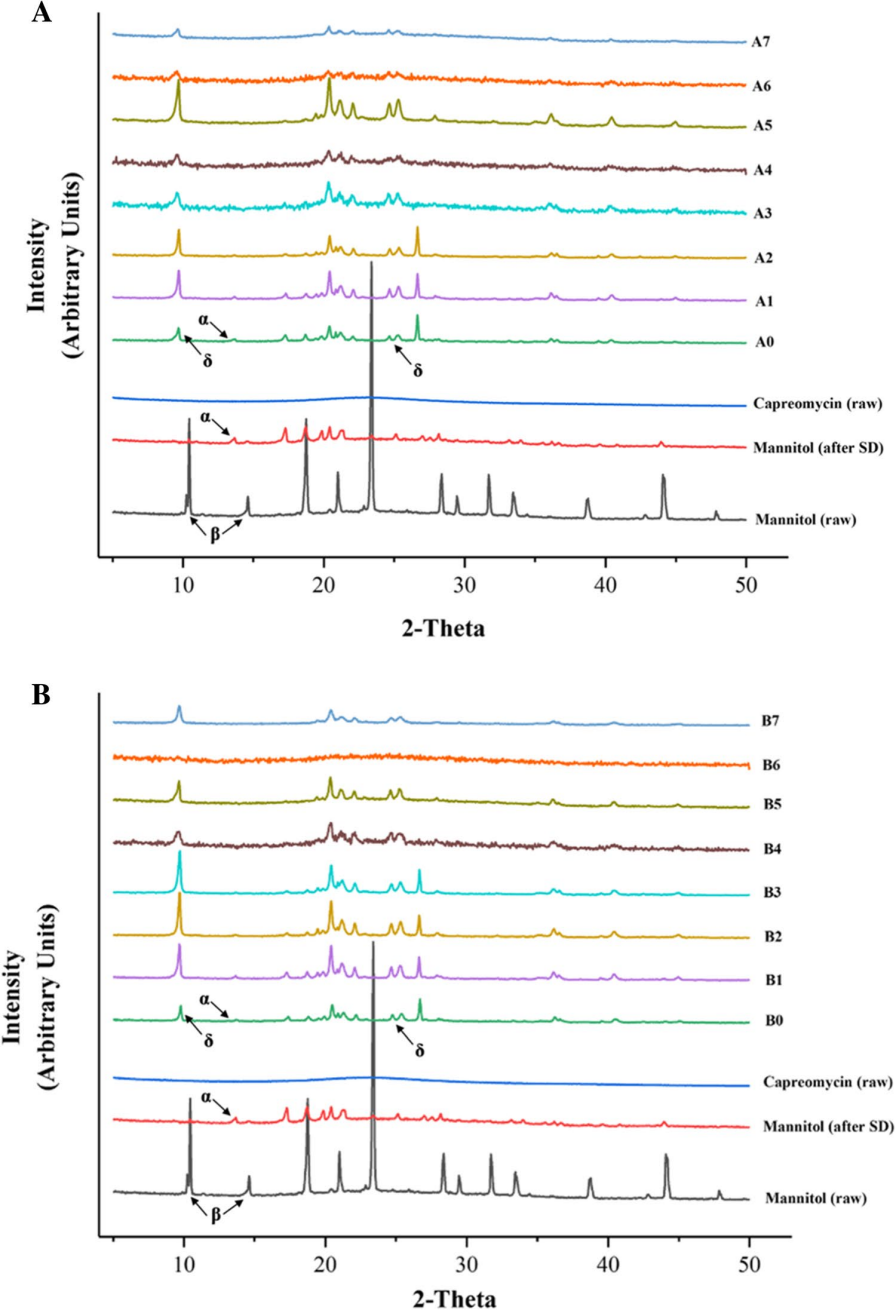
X-ray powder diffraction (XRD) diffractogram (from 5° to 50°) of (**A**) D-LAK120-A peptide and (**B**) D-LAK120-HP13 peptide containing co-spray dried powder formulations. Unformulated capreomycin (raw), unformulated mannitol (raw), and spray dried mannitol (SD) were included as controls for comparison.

**Fig. 4:**
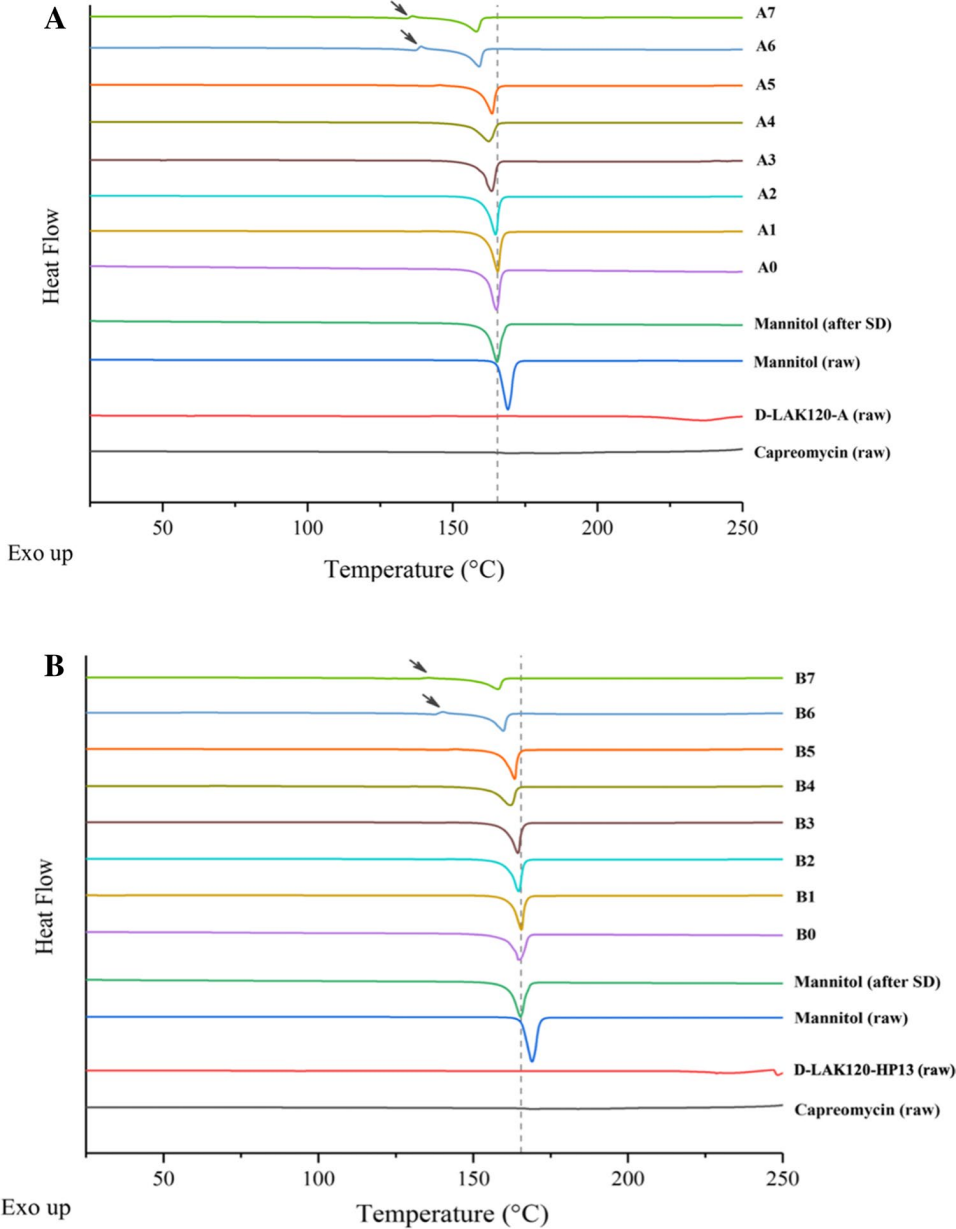
DSC thermogram of (**A**) D-LAK120-A peptide and (**B**) D-LAK120-HP13 peptide containing co-spray dried powder formulations. Negative peak represents endothermic events. The arrow points at exothermic peak in formulations A6, A7, B6 and B7. The dotted line represents 165℃. Unformulated mannitol (raw), unformulated peptide (raw), unformulated capreomycin (raw), and spray dried mannitol (SD), were included as controls for comparison.

The PXRD result (Fig. 3) showed that raw mannitol was highly crystalline, predominately in β form, as indicated by the presence of peaks at 10.56° (β) and 14.71° (β) [17]. The drug-free spray-dried mannitol was predominately in α form since it had similar diffractogram with the pure α form mannitol and the characteristic peak at 13.79° (α) [17, 18]. The capreomycin (sulfate) was amorphous without characteristic peak. In the co-spray dried powder formulations, mannitol exhibited both α and δ polymorphs, as indicated by the characteristic peak at 9.74° (δ) and the bimodal peak around 25° (δ) [17, 19]. As the proportion of capreomycin and peptide increased, mannitol exhibited δ form predominately as the characteristic peaks at 13.79° (α) diminished. Comparing all formulations, the higher the content of capreomycin and peptide, the lower the peak intensity, the lower the level of crystallinity. As shown in the DSC thermograms (Fig. 4), all samples demonstrated a characteristic endothermic peak at approximately 165 ℃, which could be interpreted as the melting point of the crystalline mannitol. As the total drug content increased, the endothermic peak shifted to the left. Interestingly, in formulations A6, A7, B6 and B7, a small endothermic peak at around 135℃ was observed (as indicated by the arrow). These formulations had a relatively high total drug content. According to the PXRD results, mannitol predominantly stayed in δ form. The δ form of mannitol tends to transit into β form when it is heated to around 130℃ [18], which could explain the small endothermic peak in formulations A6, A7, B6 and B7 in the thermogram.

### Surface Composition Analysis

**Fig. 5:**
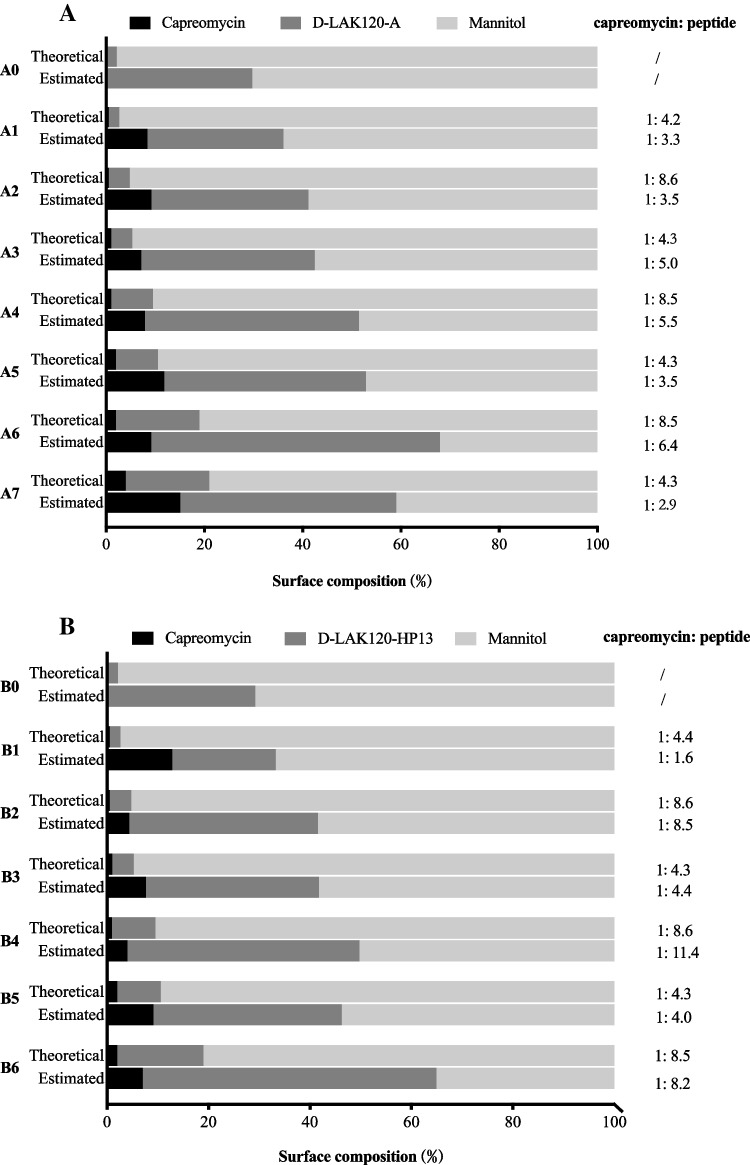
Surface composition of (**A**) D-LAK120-A peptide and (**B**) D-LAK120-HP13 peptide containing co-spray dried powder formulations evaluated by X-ray photoelectron spectroscopy (XPS). Surface composition was presented as relative atomic percentage. Theoretical (upper bar) and estimated (lower bar) surface composition of each component in the dry powder formulations were shown. (Due to the low production yield_,_ sample B7 was not analyzed).

The elemental composition of raw capreomycin, D-LAK peptides and mannitol was measured and analyzed by the number of atoms in the molecules (Supplementary Table S1). The theoretical and estimated surface compositions of capreomycin, D-LAK peptides and mannitol in the co-spray-dried powder were calculated (Supplementary Table S2) and compared (Fig. 5). Both capreomycin and D-LAK peptides showed higher estimated percentage at the surface than the theoretical value in all formulations, indicating the enrichment of capreomycin and peptide on the powder surface. In A-series, the theoretical ratio between D-LAK120-A and capreomycin was 4.2 ~ 4.3 (A1, A3, A5, A7) or 8.5 ~ 8.6 (A2, A4, A6). For formulations that had the same theoretical peptide-to-capreomycin ratio, their estimated ratio was not constant and did not show a clear trend as the total drug content increased, while doubling the peptide concentration in the formulation resulted in an increase in surface concentration of peptide. In B-series, the theoretical ratio between D-LAK120-HP13 and capreomycin was 4.3 ~ 4.4 (B1, B3, B5) or 8.5 ~ 8.6 (B2, B4, B6). The estimated ratio in formulations B2, B3, B5 and B6 was similar to the theoretical value, while that of B1 and B4 was largely deviated from the theoretical one. Akin to the A-series, the estimated ratio was neither constant nor showed obvious trend at any given theoretical ratio. In general, both capreomycin and D-LAK peptides tend to stay at the particle surface but the relative surface proportion between capreomycin and peptides fluctuated.

## Discussion

Several inhalable powder formulations of combined therapies prepared by spray drying for pulmonary TB treatment have been reported. Cunha *et al.* prepared inhalable chitosan and fucoidan microparticles containing two first-line antitubercular drugs, isoniazid and rifabutin [20, 21]. Chan *et al.* developed an excipient-free triple antibiotic dry powder formulation of pyrazinamide, rifampicin and isoniazid at therapeutically relevant ratio [22]. Momin *et al.* co-spray dried bedaquiline and pyrazinamide for latent and drug-resistant tuberculosis [23]. Pitner *et al.* co-spray dried capreomycin with CPZEN-45 into inhalable powders targeting drug-resistant tuberculosis [14]. These combination formulations, comprising exclusively of small molecules, aimed to simplify the treatment regimen substantially, enhance therapeutic efficacy, reduce dosage, improve safety profile, and most importantly, minimize risk of drug-resistance and improve patient compliance. There were only few studies on co-spray drying small molecules with macromolecules. For instance, Lindsay *et al*. co-spray dried an innate antimicrobial protein (lactoferrin) with aminoglycoside antibiotics (tobramycin or gentamicin) for pulmonary delivery against *P.aeruginosa* biofilms infections [24], focusing on the evaluation of the effects of these spray dried combinations on bacterial cell viability. There is limited research that primarily investigated the physicochemical and aerosol properties of spray dried powders containing a combination of small and large molecules, both of which are active drugs with the constraints of dose ratios.

Possessing the virtue of continuous processing, low production cost and high scalability for industrial manufacture, spray drying is a popular method in particle engineering [25]. This technique has been successful in producing inhalable powders including macromolecules such as small interference RNA (siRNA) [26], messenger RNA (mRNA) [27] and proteins [28] by optimizing various process parameters. Since the thermal and shear stresses during spray drying could potentially degrade biomolecules such as peptides and impair their integrity and hence activity, a milder processing condition should be used without compromising on other attributes such as residual moisture and production yield. The spray drying parameters used in current study for preparing combined formulations of capreomycin and D-LAK peptide were adapted from our previous studies [9, 13]. The capreomycin formulation spray-dried at 90℃ resulted in high production yield with adequate aerosol performance [13]. In another study, it was found that the D-LAK peptide could resist the thermal and mechanical stresses without noticeable degradation after spray drying at an inlet temperature of 75℃ (with an outlet temperature ranging from 40 to 42 ℃) when mannitol was used as bulking excipient. [9]. Current study chose a similar and low inlet temperature of 80℃ to avoid peptide denaturation and preserve peptides' bioactivity. However, the production yield of spray drying dropped significantly as the total drug content increased. This was probably associated with the highly amorphous particles produced in the spray drying process. It was reported that the addition of high content of protein in lactose may increase the glass-transition temperature of the overall particles, leading to a higher level of amorphicity in the particles [29]. Some protein-carbohydrate-water system studies found that the presence of protein may delay carbohydrate crystallization of particles during drying, through restricting their diffusion to form nuclei and crystal growth and inhibiting intermolecular sugar-sugar hydrogen bond formation [29–31]. Thus, this protein-carbohydrate-water system, especially with high content of protein, was probably in amorphous rubbery form during the drying process. The amorphous powder particles tend to be highly adhesive at the surface, resulting in particle–particle or particle–wall colliding during the process, therefore leading to poor production yield [29, 32–34]. According to the PXRD result, the powder formulations with high drug content, especially high peptide content, showed a lower level of crystallinity, which corresponds to a low production yield.

Morphology and surface roughness of particles can affect powder dispersibility. The particles of all formulations appeared spherical, which have a small area-to-volume ratio, making them less prone to aggregation and more readily dispersed [35]. Previously, we prepared spray dried powders containing 1%, 2% or 4% (w/w) D-LAK peptide at a higher feed rate of 3.6 mL/min and an inlet temperature of 75℃ [9]. The spray dried particles of D-LAK120-HP13 exhibited wrinkled corrugated surface while those of D-LAK120-A displayed a smooth surface [9]. This phenomenon of corrugated surface was not observed in the combination formulations containing D-LAK120-HP13, even in formulation B1 which contained only the peptide and mannitol. Maria *et. al.* reported that temperature and liquid feed rate influenced the surface roughness of spray-dried mannitol particles by affecting the size of the single crystals of mannitol that form the particle shell [36]. As current formulations contained at least 80% (w/w) of mannitol, the smoother surface of spray-dried particles was probably caused by the higher temperature and lower feed rate used during spray drying.

Apart from surface morphology, powder dispersibility is also significantly affected by particle surface composition. The tendency of surface enrichment can be affected by diffusivity, solubility, and the surface activities of the spray dried components [13, 29, 37]. The enrichment of capreomycin and peptide at the particle surface could be explained by their lower diffusivity as compared to that of mannitol, due to their higher molecular weight and lower aqueous solubility. Here, the XPS results did not reveal obvious trends between the concentrations of capreomycin and peptide in the bulk formulation and the surface composition of the spray dried particles. While both capreomycin and D-LAK peptides are water soluble, their specific aqueous solubility studies are not known, which is relevant to their diffusion rate during droplet evaporation. The complex structures of both capreomycin (which contains four molecular analogs: capreomycin IA, IB, IIA and IIB) and D-LAK peptides might also lead to interactions during droplet evaporation. Hence, the extent of surface enrichment of capreomycin and D-LAK peptides was unpredictable. This variability in surface composition was anticipated to be present in other systems involving multiple active ingredients as well, especially when they have different sizes, solubilities and surface activities, an aspect that remained to be explored.

Moreover, combination formulations with multiple active ingredients also experience additional restrictions, as the actives must be present at the relevant therapeutic dose and dose ratios, further limiting the flexibility in formulation design. A possible strategy for formulation improvement is to incorporate an excipient with high surface activity, such as leucine, to create a hydrophobic coating on the particles, thereby enhancing powder flowability and drug stability [38, 39]. A first-line anti-TB drug, rifampicin, was found to preferentially deposit on the particle surface during spray drying and constituted a hydrophobic coating in the powders, resulting in high aerosol efficiency and offering moisture protection without affecting drug dissolution [40–42]. As such, rifampicin can also be considered in this co-spray dried system as it not only enhances powder physicochemical and aerosol properties but also serves as an additional drug against susceptible *Mtb*. Nanoparticle-based inhalable powder can serve as another formulation strategy to mitigate variations in surface composition. The lipid layer or polymeric layer of nanoparticles can also provide moisture protection and the enhancement of drug stability [43].

The aerodynamic size distribution was the most decisive factor for the site of lung deposition following inhalation. Particles with an aerodynamic diameter within the range of 1–5 μm can be deposited in the lung efficiently by sedimentation [44]. Particles that are too small are readily exhaled rather than being deposited, whereas larger particles tend to undesirably retain in the inhaler or deposit at the oropharynx region through inertial impaction [36], thereby preventing particles from reaching the alveolar macrophages where mycobacteria mainly reside. In addition to primary particle size, the efficiency of particle dispersion also critically determines the deposition pattern of an inhalable powder formulation. Agglomerates of particles inside the inhaler device must be sufficiently loosened and separated during dispersion such that the effective aerodynamic diameters resemble the primary particle diameter. Hence, the relationship between airflow rate and aerosol performance was further explored by dispersing A1 and B1 at two different airflow rates of 90 L/min and 60 L/min. A lower flow rate was chosen because previous clinical studies revealed that patients with TB or TB history may have poor pulmonary function and related respiratory symptoms [45, 46], implying that they may not be able to generate sufficient inspiratory effort. Although there is no systematic analysis on the airflow rates achieved by TB patients, one study found that most chronic obstructive pulmonary disease (COPD) patients, who also suffered from compromised lung function, could achieve a flow rate of 60/L min using Breezhaler® [47]. A lower FPF was found in both formulations when the powder was dispersed at an airflow rate of 90 L/min. As expected, the dispersed particles acquired an excessive momentum at high flow rate, rendering them unable to follow the airstream upon a change in flow direction in the impactor and thus predominately deposit at the induction port and upper stages. Conversely, when a lower airflow rate of 60 L/min was used, more particles were deposited in later stages and resulted in a better FPF. This suggested that the dispersion of the dry powder formulations could be readily achieved at a lower airflow rate and dispersion pressure, whereas excessive dispersion energy could be undesirably transferred to the particles as inordinate momentum that led to premature deposition. Dry powder formulations that can be readily dispersed are preferred as TB patients with compromised lung functions may not achieve adequate airflow rate and dispersion pressure for particle deagglomeration.

Generally, dry powder for inhalation has a better stability for long-term storage compared to liquid formulation since biochemical degradation is minimized in solid state [48]. Crystallinity of solid plays an important role in the stability of powder, especially when the particles are exposed to elevated temperature and humidity in which recrystallization of amorphous solids can readily occur [49, 50]. Mannitol was used as the bulking excipient because it is safe, non-hygroscopic, non-reducing and exhibits mostly crystalline structure after spray drying. When solvent is being removed during the drying process, the sugar excipient can form hydrogen bonds with polar groups of drugs and protect them from agglomeration or denaturation [51]. Noticeably, the δ-form of mannitol, despite being the least stable crystalline form, became increasingly prominent in the presence of D-LAK peptide and capreomycin, as compared to the α- and β-form observed in spray dried mannitol and raw mannitol, respectively [52]. The protein-mannitol stabilization effect that was observed with the δ-form of mannitol in other studies [52, 53] has led to the speculation that the stability of the powder formulations is conferred in the presence of δ-mannitol, as shown previously with spray dried D-LAK peptides powders [9]. Nonetheless, the long-term stability of these combined formulations remains to be determined. Besides, the integrity and bioactivity of D-LAK peptides are crucial and will be the next step of investigation. Formulation optimization may be required to enhance the aerosol performance and production yield for formulations with high drug content. Moreover, the anti-TB activity of the combined formulations will be evaluated *in vitro* and *in vivo*. The pharmacokinetic profile will be examined in animals following pulmonary administration.

## Conclusions

Dry powder formulations containing D-LAK peptides and capreomycin, with mannitol as a bulking agent, were prepared by spray drying. Most of the co-spray dried formulations showed good production yield, low residual moisture and spherical particle shape with a smooth surface. Both capreomycin and D-LAK peptides enriched at particles surface. In the aerosol performance study using Breezhaler®, decreasing the operation flowrate to 60 L/min can improve aerosol performance with the best FPF of over 50% achieved. Further studies including formulation optimization, evaluation of peptide integrity, bioactivity and long-term stability of the powders are needed.

## Supplementary Information

Below is the link to the electronic supplementary material.

Supplementary file1 (DOCX 135 KB)

## Data Availability

Data are available upon request.
