# Supplementary material for: Co-Delivery of D-LAK Antimicrobial Peptide and Capreomycin as Inhaled Powder Formulation to Combat Drug-Resistant Tuberculosis
Source: Pharm Res. 2023 Mar 3;40(5):1073–86. doi: 10.1007/s11095-023-03488-y (PMC9984245; doi:10.1007/s11095-023-03488-y)
Supplement: Supplementary file 1 — Supplementary file1 (DOCX 135 KB) [file 11095_2023_3488_MOESM1_ESM.docx]

**Supplementary Information**

**1.1 XPS – calculation of surface composition**

Raw materials of capreomycin, two D-LAK peptides and mannitol was detected for calculation. Fluorine was found in raw D-LAK peptides, which could belong to the impurity in the D-LAK peptides. As the fluorine concentrations were around 10% in most spray-dried samples, which was high and cannot be neglected as common impurity, the fluorine was included in the calculation. Around 2% of sulfur was detected in raw capreomycin sulfate, but in most spray-dried samples, the sulfur could not be detected due to low concentration (below 0.2%). Although sulfur is a specific element of capreomycin, the low concentration may induce unexpected calculation error. Therefore, sulfur was removed during calculation. Hydrogen could not be detected by XPS and was not measured. In total, four elements (C, N, O and F) on the surface of spray-dried powders were measured and used for calculation. The calculation method was adapted from previous studies [1,2] by assuming a homogenous distribution of the components of the particle. The observed elemental concentrations were assumed to be a linear combination of the components. The regression model in matrix form with four elements (C, N, O, F) and three components can be formed as follows:

$$E_{sample}^{C}= E_{cap}^{C}\times\beta_{\mathrm{cap}} +E_{pep}^{C}\times\beta_{\mathrm{pep}} +E_{man}^{C}\times\beta_{\mathrm{man}} ①$$

$$E_{sample}^{O}= E_{cap}^{O}\times\beta_{\mathrm{cap}} +E_{pep}^{O}\times\beta_{\mathrm{pep}} +E_{man}^{O}\times\beta_{\mathrm{man}} ②$$

$$E_{sample}^{N}= E_{cap}^{N}\times\beta_{\mathrm{cap}} +E_{pep}^{N}\times\beta_{\mathrm{pep}} ③$$

$$E_{sample}^{F}= E_{pep}^{F}\times\beta_{\mathrm{pep}} ④$$

Where $E_{sample}^{C}$, $E_{sample}^{O}$, $E_{sample}^{N}$, $E_{sample}^{F}$, are the relative atomic percentage of carbon, oxygen, nitrogen and fluorine in the samples; $\beta_{\mathrm{cap}}$, $\beta_{\mathrm{pep}}$, $\beta_{\mathrm{man}}$ are the relative atomic percentage of capreomycin, peptide (D-LAK120-A or D-LAK120HP-13) and mannitol at the surface. Here, the system was overestimated and was solved by the least squares method.

**Table S1.** Theoretical and experimental percentages by the number of atoms of elements in pure capreomycin (as sulfate), two D-LAK peptides and mannitol.

| Element | Raw capreomycin  (as sulfate) | | D-LAK120-A | | D-LAK120-HP13 | | Raw mannitol | |
| --- | --- | --- | --- | --- | --- | --- | --- | --- |
|  | Theoretical | Experimental | Theoretical | Experimental | Theoretical | Experimental | Theoretical | Experimental |
| Carbon | 48.00 | 56.43 | 68.59 | 61.63 | 67.51 | 58.35 | 50.00 | 55.92 |
| Oxygen | 24.00 | 19.80 | 13.09 | 14.63 | 12.69 | 18.69 | 50.00 | 44.08 |
| Nitrogen | 28.00 | 23.77 | 18.32 | 13.16 | 19.80 | 13.52 | / | / |
| Fluorine | / | / | / | 10.58 | / | 9.44 | / | / |

| **Sample** | **Atomic percentage** | | |
| --- | --- | --- | --- |
|  | **Capreomycin** | **Peptide** | **Mannitol** |
| A0 | 0 | 29.74 | 70.26 |
| A1 | 8.39 | 27.67 | 63.94 |
| A2 | 9.20 | 31.99 | 58.80 |
| A3 | 7.11 | 35.32 | 57.58 |
| A4 | 7.88 | 43.59 | 48.52 |
| A5 | 11.85 | 41.00 | 47.15 |
| A6 | 9.15 | 58.78 | 32.07 |
| A7 | 15.05 | 44.00 | 40.95 |
| B0 | 0 | 29.19 | 70.81 |
| B1 | 12.88 | 20.38 | 66.74 |
| B2 | 4.38 | 37.22 | 58.40 |
| B3 | 7.67 | 34.11 | 58.22 |
| B4 | 4.01 | 45.77 | 50.22 |
| B5 | 9.17 | 37.09 | 53.75 |
| B6 | 7.04 | 57.89 | 35.07 |

**Table S2.** Surface composition of co-spray dried powder formulations evaluated by X-ray photoelectron spectroscopy (XPS). Surface composition was presented in relative atomic percentage. (Due to the low production yield, sample B7 was not analyzed.)

**
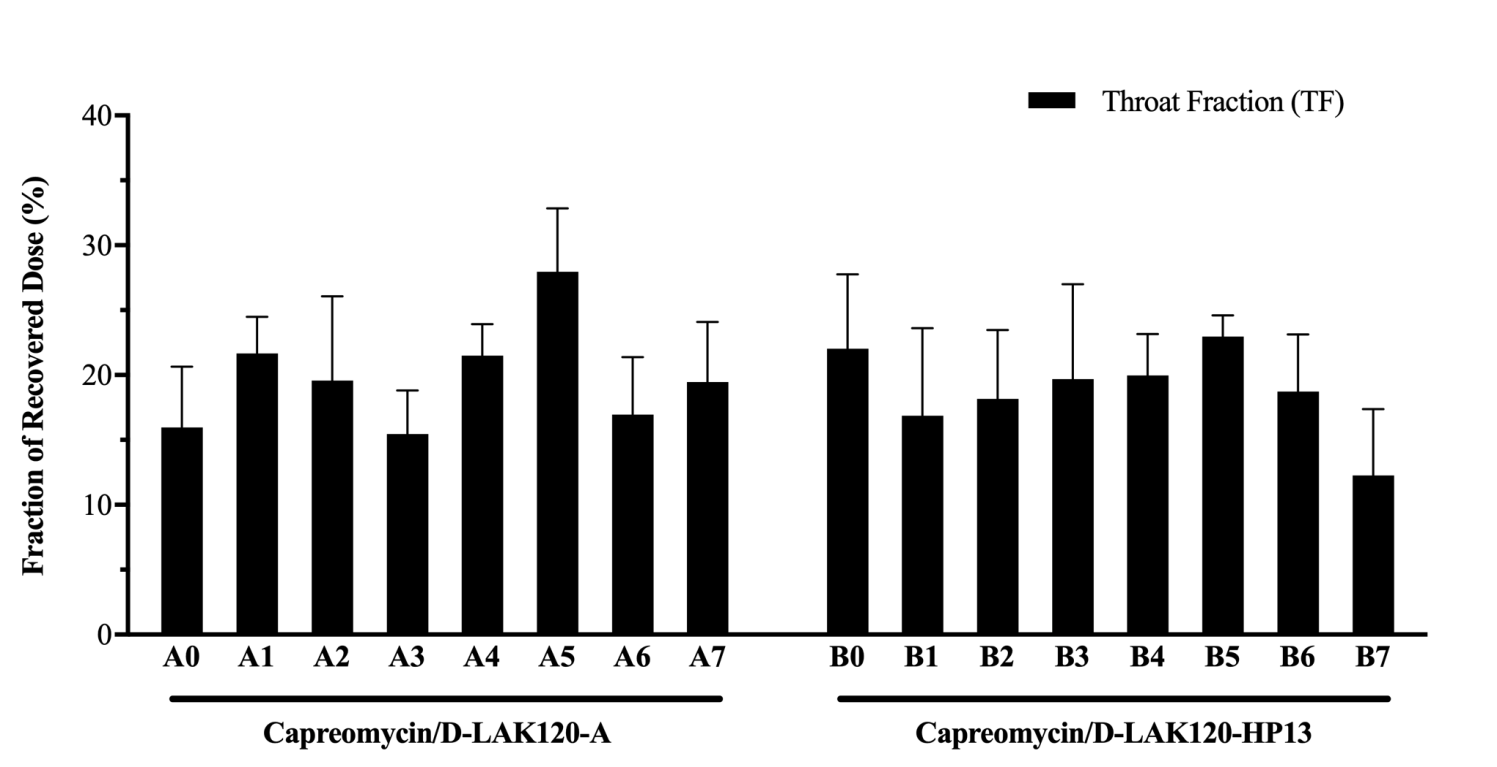
**

**Figure S1.** The throat fraction (TF) of all co-spray dried powder formulations evaluated by the Next Generation Impactor (NGI) and the powders were dispersed with Breezhaler^®^ at 90/L min. TF was expressed with respect to the recovered dose by mass. Data was presented as mean ± standard deviation (n=3).

**References**

[1] P. Faldt, B. Bergenstahl, G. Carlsson, The surface coverage of fat on food powders analyzed by ESCA (electron spectroscopy for chemical analysis), Food Structure, 12 (1993) 10.

[2] Y.-t. Chow, Development of inhaled siRNA formulation for pulmonary delivery, HKU Theses Online (HKUTO), (2018).
